# Validity and Reliability of Methods to Assess Movement Deficiencies Following Concussion: A COSMIN Systematic Review

**DOI:** 10.1186/s40798-023-00625-0

**Published:** 2023-08-14

**Authors:** Laura A. M. Dunne, Michael H. Cole, Stuart J. Cormack, David R. Howell, Rich D. Johnston

**Affiliations:** 1 School of Behavioural and Health Sciences, Australian Catholic University, Brisbane, Australia; 2 SPRINT Research Centre, Faculty of Health Sciences, Australian Catholic University, Brisbane, Australia; 3 Healthy Brain and Mind Research Centre, Faculty of Health Sciences, Australian Catholic University, Melbourne, Australia; 4 School of Behavioural and Health Sciences, Australian Catholic University, Melbourne, Australia; 5 Sports Medicine Center, Children’s Hospital Colorado, Aurora, CO USA; 6 Department of Orthopedics, University of Colorado School of Medicine, Aurora, CO USA; 7 Carnegie Applied Rugby Research Centre, School of Sport, Leeds Beckett University, Leeds, UK

**Keywords:** Sports-related concussion, Motor, Assessment, Validity, Reliability

## Abstract

**Background:**

There is an increased risk of subsequent concussion and musculoskeletal injury upon return to play following a sports-related concussion. Whilst there are numerous assessments available for clinicians for diagnosis and during return to play following concussion, many may lack the ability to detect these subclinical changes in function. Currently, there is no consensus or collated sources on the reliability, validity and feasibility of these assessments, which makes it difficult for clinicians and practitioners to select the most appropriate assessment for their needs.

**Objectives:**

This systematic review aims to (1) consolidate the reliability and validity of motor function assessments across the time course of concussion management and (2) summarise their feasibility for clinicians and other end-users.

**Methods:**

A systematic search of five databases was conducted. Eligible studies were: (1) original research; (2) full-text English language; (3) peer-reviewed with level III evidence or higher; (4) assessed the validity of lower-limb motor assessments used to diagnose or determine readiness for athletes or military personnel who had sustained a concussion or; (5) assessed the test-retest reliability of lower-limb motor assessments used for concussion management amongst healthy athletes. Acceptable lower-limb motor assessments were dichotomised into instrumented and non-instrumented and then classified into static (stable around a fixed point), dynamic (movement around a fixed point), gait, and other categories. Each study was assessed using the COSMIN checklist to establish methodological and measurement quality.

**Results:**

A total of 1270 records were identified, with 637 duplicates removed. Titles and abstracts of 633 records were analysed, with 158 being retained for full-text review. A total of 67 records were included in this review; 37 records assessed reliability, and 35 records assessed the validity of lower-limb motor assessments. There were 42 different assessments included in the review, with 43% being non-instrumented, subjective assessments. Consistent evidence supported the use of instrumented assessments over non-instrumented, with gait-based assessments demonstrating sufficient reliability and validity compared to static or dynamic assessments.

**Conclusion:**

These findings suggest that instrumented, gait-based assessments should be prioritised over static or dynamic balance assessments. The use of laboratory equipment (i.e. 3D motion capture, pressure sensitive walkways) on average exhibited sufficient reliability and validity, yet demonstrate poor feasibility. Further high-quality studies evaluating the reliability and validity of more readily available devices (i.e. inertial measurement units) are needed to fill the gap in current concussion management protocols. Practitioners can use this resource to understand the accuracy and precision of the assessments they have at their disposal to make informed decisions regarding the management of concussion.

*Trail Registration:* This systematic review was registered on PROSPERO (reg no. CRD42021256298).

**Supplementary Information:**

The online version contains supplementary material available at 10.1186/s40798-023-00625-0.

## Key Points

- Commonly used subjective static assessments such as the Balance Error Scoring System (BESS) displayed insufficient test–retest reliability and construct validity for the detection of sports-related concussion (SRC).
- Instrumented static balance assessments using laboratory equipment (i.e. force plate) or portable microtechnology (i.e. inertial measurement units) demonstrated better test–retest reliability and construct validity compared to subjective assessments. However, all static balance assessments displayed a poor ability to detect persistent symptoms of SRC beyond acute stages (> 2 weeks post).
- Instrumented dynamic assessments demonstrated sufficient test–retest reliability. The instrumented Y-balance test demonstrated sufficient sensitivity in adult populations, but poor specificity.
- Instrumented and non-instrumented gait assessments displayed sufficient test–retest reliability and construct validity. The addition of a cognitive task (dual-task) improved sensitivity.
- Laboratory assessments display sufficient reliability and validity, but poor ecological validity for the assessment of field-based sports due to the controlled environmental conditions. Associated costs, equipment, and personnel also limit the utility of these assessments for team-sport athletes.
- Clinicians are encouraged to implement instrumented or non-instrumented dynamic balance or gait assessments based on the individual needs and abilities within their setting.
- If practitioners do not have the resources to perform instrumented tests, it is recommended that they consider the reliability and validity issues that potentially limit the simpler test options, with gait assessments recommended over static or dynamic

**Supplementary Information:**

The online version contains supplementary material available at 10.1186/s40798-023-00625-0.

## Background

Concussion, otherwise referred to as mild traumatic brain injury (mTBI), is described as a transient disturbance of brain function [1] and is a common injury in contact sports, such as rugby league [2], and in certain occupations, such as military personnel [3]. Concussions are caused by transfer of energy across the brain as a result of direct (collision) or indirect (whiplash mechanism) trauma to the head and/or neck [4, 5]. Such impacts cause disruptions in normal cellular function, resulting in an ‘energy crisis’ [4–9], with symptoms typically including headache, nausea, poor coordination, vision deficits, and behavioural abnormalities such as irritability or depressive mood states [5, 10]. Given the multiple symptoms that present following a concussion, monitoring recovery can be complex for clinicians and practitioners.

To account for the multitude of symptoms experienced, a variety of assessment tools are made available for clinicians [11]. Across numerous sports, athletes diagnosed with a concussion are guided through a graduated return-to-play (RTP) process by a medical practitioner and/or rehabilitation staff. Progress through the staged RTP is primarily based upon symptom resolution at rest and during exertion as well as a return to pre-concussion baseline for cognitive and motor scores [12–15]. Of concern, however, is the ambiguity surrounding diagnostic tools and more specifically, the lack of evidence supporting their implementation in the latter stages of concussion management. For example, the common subjective balance assessments used by clinicians (e.g. BESS and tandem gait) [16] may lack the resolution to detect changes in function that can linger post-concussion. There appears to be an increased risk of subsequent concussion and musculoskeletal injuries up to 12 months following SRC [17–19], which may be linked to lingering motor deficits [20] and suggest that subclinical changes remain beyond RTP clearance that are poorly detected by many of the assessments readily available to clinicians [17, 19, 21]. Reliance on diagnostic tools as a means to evaluate recovery in conjunction with the subjective nature of many clinical assessments may explain why subtle, underlying motor changes go largely undetected [22]. Due to this concern, it is important to understand how post-concussion changes in motor performance can be monitored more effectively, thus allowing clinicians to make decisions based on sound objective data as well as clinical judgement.

To minimise the risk of incorrect recovery diagnosis, assessments need to demonstrate clinically acceptable reliability and validity, whilst also being feasible to conduct. Reliability refers to an instrument’s ability to produce consistent measures across multiple time points, thus ensuring change in score is attributed to changes in performance as opposed to instrument errors [23, 24]. Validity can be broken into three categories; logical, criterion, and construct [25]. For this review, only construct validity has been reported, i.e. an instrument’s ability to correctly diagnose concussed and non-concussed populations. The higher the sensitivity and specificity of an instrument, the better its ability to classify those with and those without concussion [25]. Feasibility is also vital to consider when selecting a test, the time, and the resources and expertise required as these will influence which tests can be administered.

Numerous lower-limb motor assessments are reported in the literature to monitor impairments following concussion, with varying time, expertise and equipment requirements. Despite this, there is no consensus or collated sources on the reliability, validity and feasibility of these assessments, which makes it difficult for clinicians and practitioners to select the most appropriate assessment based on needs and time since concussion. This systematic review aims to [1] consolidate the reliability and validity of motor function assessments across the time course of concussion management and [2] summarise their feasibility for clinicians and other end-users. The purpose is to provide clinicians with evidence to support the utility and practicality of selected assessments and identify potential gaps in the current management of concussion.

## Methods

### Search Strategy

This systematic review was structured in accordance with the Preferred Reporting Items for Systematic Reviews and Meta-Analyses (PRISMA) statement [26] and registered on PROSPERO (reg no. CRD42021256298). Five academic databases, including SPORTDiscus, CINAHL, Web of Science, Medline, and Scopus were systematically searched from earliest record to May 17, 2023. Eligible studies were identified through searching titles, abstracts, and keywords for predetermined search terms (Table 1). References were extracted from each database and imported into a reference manager (EndNote X20, Clarivate Analytics, London, United Kingdom) before removing any duplicate articles. Subsequently, to allow simultaneous, blinded screening, articles were imported into Covidence (www.covidence.org; Melbourne, Australia), an online tool for systematic reviews. Titles and abstracts were analysed by one reviewer (LD); the full texts of the remaining studies were then assessed by two reviewers (LD and RJ). Where any conflicts arose, the two reviewers met to determine study eligibility.

**Table 1:** Search terms used for review; search 1 to 5 was combined with the operator ‘AND’, search 6 was combined with the operator ‘NOT’

Search 1	sport OR athlete OR player* OR military OR soldier OR “service men” OR “service member*”
Search 2	concuss* OR "sports related concussion" OR "sports-related concussion" OR mTBI OR "mild traumatic brain injury" OR "sport-induced concussion" OR "sport induced concussion" OR "mild head injury"
Search 3	assessment* OR test* OR evaluat* OR analysis OR examination OR outcome OR measure
Search 4	COP OR centre of pressure OR center of pressure OR gait OR movement OR single task OR single-task OR stiffness OR motor OR neuromuscular OR IMU OR "inertial measurement unit” OR accelerom* OR landing OR dynamic balance
Search 5	validity OR reliability OR sensitivity OR specificity OR “test–retest reliability”
Search 6	“motor accident” OR “car accident” OR “car crash” OR “wreck” OR “vehicle accident” OR “vehicle crash” OR “truck crash”

### Eligibility Criteria

Eligible studies must have (1) been original research articles (2); been full-text articles written in the English language (3); been peer-reviewed articles with level of evidence equal to or greater than level III [27]; (4) assessed the validity of lower-limb motor assessments used to diagnose or determine RTP readiness for athletes or military personnel who had sustained a concussion or (5); assessed the test–retest reliability of lower-limb motor assessments used for concussion management amongst healthy athletes. Acceptable lower-limb motor assessments were classified into four categories: static, dynamic, gait, and other. Static balance assessments included tasks in which individuals remained in a fixed point during various stances (e.g. BESS) where postural sway or number of balance errors were the outcome variables. Dynamic balance assessments included any task that required movement (e.g. limb excursion) from an individual, while remaining at a fixed point (e.g. Y-balance test). Gait assessments comprised of any task that required locomotion with both temporal and/or spatial parameters measured. Assessments that were specific for sport or military tasks were categorised as other. Further categorisation was performed with assessments being classified as non-instrumented (subjective scoring or use of basic equipment [i.e. Stopwatch]) or instrumented (objective [i.e. accelerometers]).

For studies to be included as reliability studies, they must have assessed the test–retest (intra-class correlation coefficient (ICC)) or inter-rater reliability of an assessment in healthy athletes. For validity, studies must have assessed the between-group differences of a lower-extremity motor task in a case–control study or shown the predictive performance of the measure to diagnose concussed and healthy participants (i.e. area under the curve (AUC), sensitivity, specificity). Reference lists from eligible studies were manually examined for any studies missed during initial search. Selected studies were then screened and assessed for eligibility. Commentaries, letters, editorials, conference proceedings, case reports, conference abstracts, or non-peer-reviewed articles were excluded. Studies examining animal or biomechanical models of brain injury were also excluded from analysis.

### Data Extraction and Analysis

Data from eligible studies were extracted into Covidence. Data pertaining to study characteristics and protocols were first extracted from eligible studies. All relevant outcome measures (reliability and/or validity measures) were extracted from each study. Data were categorised according to: assessment type (e.g. static, dynamic, gait) and relevant findings being reliability and/or validity (e.g. sensitivity, specificity). Due to the heterogeneous nature of the findings, a meta-analysis was not performed.

### Quality Assessment

To assess the methodological quality and the clinical reported outcome measurements (ClinROMs; reliability and validity), the Consensus-based Standards for the selection of health Measurement INstruments (COSMIN) Risk of Bias tool for outcome measurement instruments [28] and the COSMIN guideline on Risk of Bias to assess quality of studies on reliability and measurement error, that is the variability between repeated measures, were used [29]. The COSMIN checklists were developed to quantitatively assess the methodological quality of studies and the ClinROMs evaluated. The first step involved rating the methodological quality for each study, which was assessed against nine measurement properties: content validity, internal structure (structural validity, internal consistency, and cross-cultural validity), reliability, measurement error, criterion validity, hypotheses testing for construct validity, and responsiveness. Each measurement property was assessed using a four-point grading scale; v*ery good*, where the model or formula was described and matched the study design; *adequate*, where the model or formula was not described, or did not match the study design; *doubtful*, where no evidence of systematic difference was provided; and *inadequate*, where calculation was deemed not optimal. Overall methodological reporting quality was determined using the ‘worst score counts’ approach [28, 30]. Feasibility of the assessment tool is no longer included within COSMIN’s measurement properties as it does not refer to the quality of an outcome measurement instrument. We highlighted the feasibility of an instrument, by reporting the interpretability of the outcome, time to complete, and equipment and expertise required. The second step was to rate the ClinROMs from each study (validity and/or reliability values) using the COSMIN criteria for good measurement properties guideline [30]. A rating of *sufficient* ( +), *insufficient* (−), or *indeterminate* (?) was given for each assessment’s measurement property based on the statistical outcome measures for each measurement property [29]. Two authors (LD and RJ) independently assessed the methodological quality and measurement property of all studies; any disagreements were discussed by these authors.

## Results

### Search Results

**Fig. 1:**
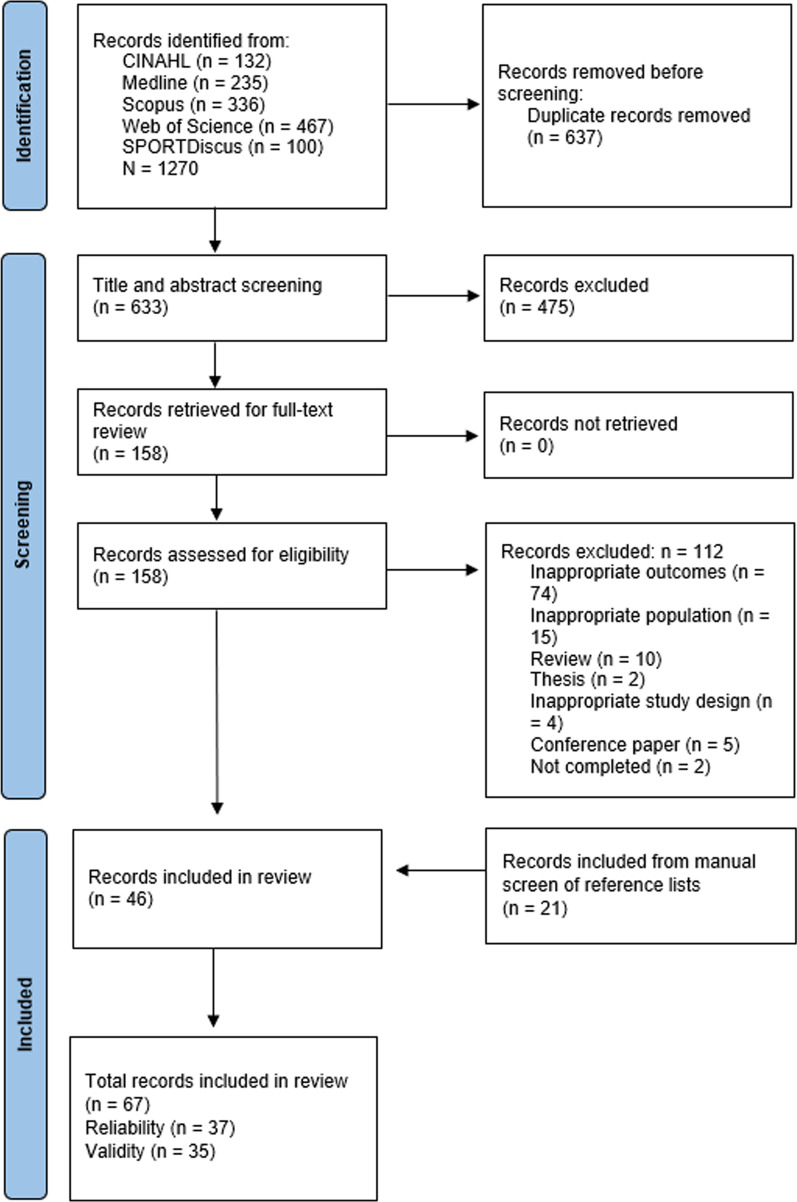
PRISMA flowchart depicting steps taken in the search strategy

The systematic search retrieved 1270 results from five academic databases, of which 637 duplicates were removed. Titles and abstracts of the remaining 633 studies were screened, with 475 not meeting eligibility criteria. Full-text review was conducted on the remaining 158 studies, with 112 deemed ineligible. A total of 46 studies were eligible, with an additional 21 included via the manual screening of reference lists. Therefore, this review included a total of 67 studies. The identification process is outlined in Fig. 1.

### Research Quality

The quality of research investigating the reliability and/or validity of lower-limb motor assessments for concussion management was variable, with methodological reporting quality ranging from *inadequate* to *very good*. Measurement property quality for all studies ranged from *sufficient* to *indeterminate* (see Additional file 1: Tables S1–S18).

#### Study Characteristics

##### Reliability

Studies were conducted on healthy adults (*n* = 29) and minors (*n* = 8), with a total sample size of 6888. The most common assessments were the BESS and tandem gait (instrumented and non-instrumented), each representing 15% of all assessments. A summary of study characteristics is presented in Tables 2, 3, 4 and 5. A full table of study characteristics is presented in Additional file 1: Table S1 through to Additional file 1: Table S7.

**Table 2:** Overview of reliability, validity and measurement error for static balance assessments for assessments used to monitor movement changes following a concussion

	Time taken	Equipment	Test procedure	Outcome variable	Reliability	Validity	Measurement error	Optimal conditions
Non-instrumented static assessments								
*Balance error scoring system (BESS)*								
[33, 34, 37–39, 42–44, 50, 51]	2 trials, 4 conditions: 5 min	Stopwatch Foam pad	Double leg, single leg and tandem stance on firm surface and foam Number of errors each 30-s stance recorded	Number of errors	*r* = 0.41 to 0.94 Inter-tester: *r* = 0.20–0.96	Sens = 0.23 to 0.60 Spec = 0.50 to 0.92 AUC = 0.51 to 0.63	MDC test–retest = 7.3 errors MDC inter-tester = 9.4 errors	3 examiners, minimum of 2 trials on 4 conditions (excluding double leg stances)
*Modified balance error scoring system (mBESS)*								
[33, 39, 40, 42–44, 52–58]	~ 2–5-min	Stopwatch	Double leg, single leg, tandem stance on firm surface only Number of errors each 30-s stance recorded	Number of errors	*r* = 0.02–0.88 Inter-tester: * r* = 0.80–0.83	Sens = 0.05–0.71 Spec = 0.0.63–0.85 AUC = 0.54–0.72 Between-group differences: p = < 0.001–0.23	–	Minimum of 2 trials Familiarisation required for children
*Single leg stance (SLS)*								
[59, 60]	~ 2–5-min	Stopwatch	Errors recorded in 30-s stance period	Number of errors	*r* = 0.85	–	–	Most participants reached 30-s cut off easily
*Modified clinical test of sensory interaction in balance (MCTSIB)*								
[56]	~ 5–10-min	NA	Double leg stance; stable surface eyes open, stable surface eyes closed, unstable surface eyes open, unstable surface eyes closed	Number of errors	–	Sens = 0.37 Spec = 0.88	–	Poor sensitivity approximately 2 weeks post-concussion
*Paediatric clinical test of sensory interaction in balance (PCTSIB)*								
[56]	~ 5–10-min	NA	Double leg stance; stable surface eyes open, stable surface eyes closed, unstable surface eyes open, unstable surface eyes closed	Number of errors	–	Between-group differences: p = 0.46–0.49	–	
Instrumented static assessments								
*Instrumented balance error scoring system*								
[32, 38, 44, 45, 51]	~ 5-min	Force plate or IMU	BESS performed on force plate (foam pad placed on plate)	Number of errors, COP or COM displacement	*r* = 0.88–0.89	Sens = 0.38–0.0.94 Spec = 1 AUC = 0.70	SEM = 0.00–0.45	Perform in first month post-concussion to maximise sensitivity
*Instrumented modified balance error scoring system*								
[44, 57, 61–63]	~ 2–5-min	Force plate or IMU	MBESS on force plate or with IMU	Number of errors, COP or COM displacement	Force plate: * r* = 0.69–0.77 Smartphone: * r* = 0.66–0.75	Sens = 0.42–0.92 Spec = 0.76 -1.00 AUC = 0.74–0.91	–	
*Instrumented single leg stance (instrumented SLS)*								
[64]	~ 2–5-min	Force plate	Measure of COP on force plate Eyes open and eyes closed conditions	Number of errors, COP displacement	*r* = 0.58–0.88		SEM = 0.41–2.97	Eyes closed condition displayed better reliability
*Balance accelerometry measure (BAM)*								
[23, 65, 66]	~ 5–10-min	IMU, smartglasses, or smartphone	6 stances: firm surface—double leg eyes open, double leg eyes closed, tandem eyes open, tandem eyes closed Foam surface—double leg eyes open, double leg eyes closed Number of errors each 45-s recorded	Number of errors, COM displacement	*r* = 0.28–0.86	AUC < 2 weeks = 0.74–0.77 AUC > 2 weeks = 0.67–0.68	-	Perform within 2 weeks post-concussion
*Balance tracking system*								
[31, 67]	~ 2-min	Force plate	4 × 20-s trials: 1 familiarisation and 3 recorded. Participant standing on force plate with eyes closed and hands on hips COP recorded	COP displacement	–	Sens = 0.64	–	Baseline scores better for comparison
*SWAY balance*								
[68]	~ 2–5-min	Smartphone	5 × 10-s stances: bipedal, tandem (R), tandem (L), single leg (R), single leg (L) on firm surface with eyes closed SWAY scores calculated	COM displacement	*r* = 0.47–0.86	–	SEM = 5.77–7.56	3 sessions Mobile held on sternum
*Sensory organisation test (SOT)*								
[69–72]	~ 10–20-min	SOT laboratory equipment	6 conditions: stance eyes open, eyes closed, eyes open with visual interference, eyes open with surface interference, eyes closed with surface interference, eyes open with visual and surface interference Each lasting 20-s	COP displacement	*r* = 0.3–0.72	Sens = 0.13–0.73 Spec = 0.85–0.95 AUC = 0.79	SEM = 4.92–5.00	
*Physiological vibration acceleration (Phybrata) System*								
[46]	~ 2-min	IMU	20-s stance with eyes open, and then eyes closed	COM displacement	–	Sens = 0.92 Spec = 0.94 AUC = 0.98	–	
*Instrumented modified clinical test of sensory interaction in balance (MCTSIB)*								
[73]	~ 5–10-min	Force plate	Double leg stance; stable surface eyes open, stable surface eyes closed, unstable surface eyes open, unstable surface eyes closed	Number of errors, COP displacement	–	Sens = 0.37 Spec = 0.88	–	
*Virtual reality balance*								
[47, 74]	~ 10-min	Force plate Virtual reality system	Standing in tandem stance, hands on hips. Participant placed in harness and positioned in 3D virtual reality room. 10 trials performed, 1 with the room still, 9 with it moving. Each trial lasted 30-s	COP displacement	–	Sens = 0.86 Spec = 0.88 AUC = 86 Between-group differences: *p* = 0.006- < 0.001	–	10 trials–1 trial with still room, 9 trials with rotating room

**Table 3:** Overview of reliability, validity and measurement error for dynamic motor assessments used to monitor movement changes following a concussion

	Time taken	Equipment	Test procedure	Outcome variable	Reliability	Validity	Measurement error	Optimal conditions
Non-instrumented dynamic assessments								
*Clinical reaction time*								
[50]	~ 3-min	Pole	Stick drop test: converted to speed	Distance on stick, speed	*r* = 0.32	–	–	
*Kasch pulse recovery (KPR)*								
[48]	~ 5-min	NA	3-min stair climb at rate of 24-steps.min Exercise HR and HR recovery (1-min seated) recorded	HR pre, during, and post	–	Sens = 1.00 Spec = 0.96 AUC = 0.98	–	Performed on children
*Physical and neurological examination of subtle signs (PANESS)*								
[75]	~ 10–15-min	Stopwatch	Gait: heel walk, toe walk, walk on side of feet, tandem gait Stations: 20-s stance (a) feet together, eyes closed, arms out, (b) eyes closed, tongue protruding, (c) tandem stance, hop each foot (50 hops)	Number of errors	–	Sens = 0.76 Spec = 0.90	–	
Instrumented dynamic assessment								
*Instrumented Y balance test (instrumented YBT)*								
[35, 76]	~ 5-min	IMU	Standing on 1 leg, tap toe of non-standing leg in anterior, posterior-medial, and posterior lateral directions	Reach distance, COM displacement	*r* = 0.76–0.99	Sens = 0.76 Spec = 0.53	–	Improved validity with use of lumbar IMU
*Instrumented limits of stability test (instrumented LOS)*								
[36, 77]	~ 5–10-min	Force plate	8 trials shifting centre of gravity in directions: forward, right/forward, right, right/back, back, left/back, left, left/forward	COP displacement	*r* = 0.973–0.96	–	SEM = 0.35–1.17	
*Dynamic postural stability index (DPSI) and DPSI with dual task*								
[64]	~ 2–5-min	Force plate	3 trials of jumps: forward, lateral, and 90° rotation	COP during landing	DPSI: * r* = 0.58–0.93 DPSI DT: * r* = 0.32–0.80	–	DPSI SEM = 0.0047–0.023 DPSI + DT SEM = 0.004–0.019	Improved reliability with standard DPSI
*Postural stress test (PST)*								
[78]	~ 5–10-min	Pulley system	Pulley system providing external perturbations. Participant required to maintain upright position. Amount of weight required to cause stepping postural adjustment is recorded	Amount of weight required to maintain balance	–	Between-group differences: *p* = 0.027–0.024	–	
*Computer-assisted rehabilitation environment (CAREN) system*								
[79]		Accelerometry, motion capture	Virtual reality standing and walking with external perturbations	COM displacement	–	Sens = 0.65–0.90	–	Walking with perturbations more sensitive 10 trials performed
*Battery combined: grip strength, single leg jump, MCTSIB*								
[73]	~ 10–15-min	Force plate, tape measure, handheld dynamometer	Grip strength using handheld dynamometer Standing long jump: participants to jump maximum distance off a single leg MCTSIB: Double leg stance; stable surface eyes open, stable surface eyes closed, unstable surface eyes open, unstable surface eyes closed	Number of errors, COP displacement, grip strength	–	Sens = 0.41 Spec = 0.77	–	Individual measures of MCTSIB, grip strength, and standing long jump displayed poor sensitivity Higher sensitivity with combined score
*Bruininks–Oseretsky test of motor proficiency*								
[78]	15–60-min total Balance component includes 8 items ~ 15-min	Requires specialised equipment	Gross motor: running speed and agility, balance, bilateral coordination, strength Fine motor: response speed, visual motor control, upper-limb dexterity	Number of errors, time to complete trials	–	Between-group differences: p = 0.024–0.001	–

**Table 4:** Overview of reliability, validity and measurement error for gait assessments used to monitor movement changes following a concussion

	Time taken	Equipment	Test procedure	Outcome variable	Reliability	Validity	Measurement error	Optimal conditions
Non-instrumented gait assessments								
*Community balance and mobility scale*								
[80, 81]	~ 10–15-min	Stopwatch, rigid box, 1–3-kg weight	Tasks: unilateral stance, tandem walk, 180° tandem pivot, lateral foot scooting, hopping forward, crouch and walk, lateral dodging, walk and look, run with stop, forward/backward walk, walk, look and carry, descend stairs, step-ups Scoring on scale 0–5	Number of errors, time to complete	–	Sens = 0.78–1.00 Spec = 0.88–0.91 AUC = 0.92–0.98	–	Performed on adult population Perform within 2 weeks post-concussion
*Timed up and go (TUG)*								
[82]	1–2-min	Chair Tape measure	Seated in chair, participant stands and walks 3 m, turns 180° and sits back down	Time to complete, errors	*r* = 0.85 Inter-tester: * r* = 0.99	–	–	
*Walking on balance beam*								
[82]	1–2-min	Balance beam, stopwatch	Participants walk on balance beam	Number of errors	*r* = 0.87 Inter-tester: * r* = 0.35	–	–	
*Tandem gait*								
[43, 52, 54, 55, 58–60, 82–84]	2–5-min	Stopwatch, measuring tape	Heel to toe walking	Time to complete, number of errors	*r* = 0.46–0.98 Inter-tester: * r* = 0.70	Sens = 0.63–0.88 Spec = 0.61–0.72 AUC = 0.55–0.86 Between-group differences: *p* = < 0.001	SEM = 0.07	
*Dual task tandem gait*								
[54, 58, 83, 84]	2–5-min	Stopwatch, measuring tape	Heel and toe walk with addition of cognitive task (count back in 7’s, recite alphabet backwards etc.)	Time to complete, number of motor and cognitive errors	*r* = 0.84–0.92	Sens = 0.85 Spec = 0.72 AUC = 0.80–0.87 Between-group differences: *p* = 0.002- < 0.001	SEM = 0.06	Cognitive tasks need to be age-specific
*Complex tandem gait*								
[56]	2–5-min	Stopwatch, measuring tape	Normal gait with addition of cognitive task (count back in 7’s, recite alphabet backwards etc.)	Time to complete, number of errors	–	Sens = 0.41 Spec = 0.90	–	Cognitive tasks need to be age-specific
*Gait*								
[58]	2–5-min	Stopwatch, measuring tape	Normal gait	Time to complete	–	Between-group differences: *p* = 0.006	–	
*Dual task gait*								
[58, 85]	2–5-min	Stopwatch, measuring tape	Normal gait with addition of cognitive task (count back in 7’s, recite alphabet backwards etc.)	Time to complete, number of motor and cognitive errors	–	Sens = 0.77 Spec = 1.00 Between-group differences: *p* = 0.29	–	Cognitive tasks need to be age-specific
Instrumented gait assessments								
*Battery combined: berg balance, balance evaluation systems Test (BESTest), dynamic gait index, high-level mobility assessment tool (HiMat)*								
[79]	~ 60-min	HiMat, tape measure, step, chair, ramp, obstacle	Berg balance: sit to stand, stand, sit, stand to sit, transfer, stand with eyes closed, stand with feet together, reach forward, reach to floor, turn to look behind, turn 360°, step, tandem stance, single leg stance BESTest: 36 items assessing bio-mechanical constraints, stability limits, anticipatory response, postural response, sensory orientation, stability in gait DGI: 8 items—walking on level surface, changing speed, head turn horizontal, head turn vertical, 180° turn, step over, step around, stair ascent, descent HiMat: 13 items—walk, walk backward, walk on toes, over obstacle, run, skip, hop, bound, stair ascent, descent	Number of errors, time to complete	–	Score ‘no impairment’ = 89% concussed	–	10 trials
*Battery combined: gait, tandem gait, dual-task tandem gait*								
[58]	~ 10–20-min	Smartphone	Gait: assessing cadence and velocity (instrumented) Tandem gait: assessing time to complete (non-instrumented) Dual-task tandem gait: assessing time to complete (non-instrumented)	Time to complete, COM displacement	–	AUC = 0.91	SEM = 0.05	Combined score displayed highest AUC
*Instrumented dual task tandem gait*								
[86, 87]	2–5-min	IMU	Heel to toe walk with addition of cognitive task (count back in 7’s, recite alphabet backwards etc.)	COM displacement, accelerometer metrics, number of cognitive errors	*r* = 0.73–0.94	–	SE = -0.080–1.355	Cognitive tasks need to be age-specific
*Instrumented gait*								
[58, 86–94]	2–5-min	IMU, 3D motion capture, GAITRite, smartphone, virtual reality	Normal gait	Time to complete, COM or COP displacement	*r* = 0.10–0.99 Internal consistency: * r* = 0.76–0.97	Sens = 0.26–0.63 Spec = 0.62–0.85 AUC = 0.76–0.79 Between-group differences: *p* = 0.99–0.002	MDC = 0.875–7.42 SEM = -1.32–0.08	
*Instrumented dual task gait*								
[58, 91–95]	2–5-min	IMU or 2D motion analysis	Normal gait with addition of cognitive task (count back in 7’s, recite alphabet backwards etc.)	COM or COP displacement, time to complete, number of motor and cognitive errors	Internal consistency: * r* = 0.61–0.97	Sens = 0.26–0.70 Between-group differences: *p* = 0.93–0.07	MDC = 0.839–6.76	Cognitive tasks need to be age-specific
*Functional gait assessment*								
[80, 81]	5–20-min	Stopwatch, measuring tape, 22 cm high obstacle, stairs	10 items; 6-m gait, 6-m gait alternating speed, gait with horizontal head turns, gait with vertical head turns, gait and pivot turn, step over obstacle, gait with narrow base of support, gait with eyes closed, backwards gait, ascend/descend stairs	Time to complete, number of errors	–	Sens = 0.05–0.88 Spec = 0.75–1.00	–	Performed on adult population

**Table 5:** Overview of reliability, validity and measurement error for other motor assessments used to monitor movement changes following a concussion

	Time taken	Equipment	Test procedure	Outcome variable	Reliability	Validity	Measurement error	Optimal conditions
Non-instrumented task specific assessment								
*Run-roll-aim*								
[41]	10–20-min	NA	4 trials, military course with combat specific tasks including roll and aim	Time to complete, number of errors	Inter-tester: * r* = 0.28–0.89	Between-group differences: *p* = < 0.01	–	Military specific
*Portable warrior test of tactile agility*								
[96]	10–20-min	Stopwatch	5 trials, military course with combat specific tasks including run, roll, and backwards run. Performed under single task and dual-task conditions	Time to complete, number of errors	–	Between-group differences single-task: *p* = < 0.001 Between-group differences dual-task: *p* = 0.004	–	Military specific
Instrumented task specific assessment								
*Instrumented portable warrior test of tactile agility*								
[49]	10–20-min	IMU	5 trials, military course with combat specific tasks including run, roll, and backwards run. Performed under single task and dual-task conditions	Time to complete, number of errors, COM or COP displacement	–	AUC of ‘lowering and rolling’ movements: AUC = 0.83 Between-group differences: 0.08–< 0.0001	–	Military specific

There were 22 different lower-limb motor assessments used across 37 different studies; 12 studies assessed the reliability of more than one test; and one study assessed reliability for adults and minors (see Additional file 1: Table S1). Assessments were categorised as static balance (*n* = 20 studies, 9 different assessments), dynamic balance (*n* = 5 studies, 4 different assessments), gait (*n* = 13 studies, 9 different assessments), or other (*n* = 1 study, 1 assessment). Studies were further subdivided based on type of reliability: test–retest (*n* = 34 studies, 20 different assessments) or inter-rater (*n* = 5 studies, 5 different assessments) and instrumented (*n* = 13 assessments) or non-instrumented (*n* = 9 assessments).

##### Static Balance Assessments

For static balance assessments, test–retest correlations ranged from 0.13 to 0.94 with measurement property quality ranging from *doubtful* to *adequate*. Outcome variables for non-instrumented assessments included time and number of errors. Instrumented assessments reported number of errors, centre-of-mass (COM) displacement, and centre-of-pressure (COP) displacement. Time between assessments ranged from the same day to 20 months, with a tendency for poorer reliability over longer periods. Assessments included BESS (*n* = 5), instrumented BESS (*n* = 2), modified BESS (mBESS) [double leg, single leg, and tandem stance on firm ground] (*n* = 3), instrumented mBESS (*n* = 1), single leg stance (*n* = 2), instrumented single leg stance (*n* = 1), double leg balance using accelerometers (balance accelerometry measure (BAM)) (*n* = 2), double leg balance on a portable force plate (balance tracking system) (*n* = 1), double- and single-leg balance (SWAY balance mobile application) (*n* = 1), and the Sensory Organization Test (SOT) (*n* = 2). The BESS demonstrated *sufficient* reliability when conducted with one trial (ICC = 0.60–0.78). However, reliability was improved when double leg stance was removed and 2–7 trials were performed (ICC = 0.83–0.94). Instrumented BESS using a force plate and Wii Balance Board (0.88–0.89) and the balance tracking system (ICC = 0.92) also displayed *sufficient* reliability over seven- and 15-day periods, respectively [31, 32]. The BESS and mBESS showed improved reliability with increased number of trials [33]. It is imperative to note that, while studies report improved reliability with increased number of trials, these assessments are routinely performed only once in clinical practice. In summary, a minimum of 2-trials on 4 conditions (excluding double leg variations) of the BESS displayed *sufficient* test–retest reliability over a seven day period [34]. The balance tracking system utilising a force plate also displayed *sufficient* reliability in addition to offering clinicians more in-depth, objective analysis [31].

##### Dynamic Balance Assessments

For dynamic balance assessments, test–retest correlations ranged from 0.32 to 0.99, with measurement property quality ranging from *doubtful* to *adequate*. Outcome variables included time, number of errors, COM displacement, and COP displacement. Time between assessments ranged from same day to 11 months, with a median of seven days, with a tendency for poorer reliability over periods greater than 10-days. Assessments included instrumented Y-balance test (*n* = 1), clinical reaction time (*n* = 1), instrumented limits of stability test (*n* = 2), and the dynamic postural stability index (DPSI) (*n* = 1). The most reliable assessments were the instrumented Y-balance test (ICC = 0.76 to 0.99), which performed same-day test–retest reliability [35] and the instrumented limits of stability test (ICC = 0.95 to 0.96), with tests conducted seven days apart [36]. Both assessments provided clinicians with consistent objective measures across trials.

##### Gait Assessments

For gait assessments, test–retest correlations ranged from 0.10 to 0.99, with measurement property quality ranging from *doubtful* to *adequate*. Outcome variables for non-instrumented assessments included time or number of errors. Instrumented assessments reported COM displacement, COP displacement, and spatio-temporal metrics. Time between assessments ranged from same day to 11 months, with a median of seven days and a tendency for poorer reliability over periods greater than two weeks. Assessments included tandem gait (*n* = 6), instrumented gait (*n* = 7), instrumented dual-task gait (*n* = 2) dual-task tandem gait (*n* = 2), instrumented dual-task tandem gait (*n* = 2), timed up and go (TUG) (*n* = 1), and walking on a balance beam (*n* = 1). Most gait assessments displayed *sufficient* test–retest reliability; however, non-instrumented assessments displayed *insufficient* reliability across periods extending greater than two months. Instrumented gait assessments (e.g. normal, tandem, and dual task gait) utilising force plates or inertial measurement units (IMU) were most consistent across time points extending to eight months. Outcome variables including step length, step time, and gait velocity were most reliable.

##### Inter-Rater Reliability

Correlations for inter-rater reliability of non-instrumented assessments performed on healthy controls ranged from 0.20 to 0.99, with measurement property quality *adequate* for all studies. Static balance assessments included BESS (*n* = 4), which ranged from 0.20 to 0.96 when using 3 assessors [32, 37, 38], and mBESS (*n* = 2), with reliability ranging from 0.80 to 0.83 using 2 and 3 assessors, respectively [39, 40]. Gait assessments included tandem gait (*n* = 1), TUG (*n* = 1), and walking on balance beam (*n* = 1). The TUG demonstrated best inter-rater reliability (ICC = 0.99) amongst two assessors. Other assessments consisted of the military-specific task run-roll-aim (*n* = 1), with reliability ranging from 0.28 to 0.89 [41].

##### Validity

The validity of 32 different assessments was reported across 35 studies; 17 studies assessed the validity of more than one test. Assessments were categorised into static balance (*n* = 21 studies, 13 different assessments), dynamic balance (*n* = 8 studies, 8 different assessments), gait (*n* = 13 studies, 8 different assessments), or other (*n* = 3 studies, 2 different assessments), and analysed either construct (*n* = 30) or known-group validity (*n* = 8 studies). Studies were conducted on adults (*n* = 24) and minors (*n* = 11), with a total sample size of 1417 concussed and 1616 control participants. A summary of study characteristics is presented in Tables 2, 3, 4 and 5. A full table of study characteristics is presented in Additional file 1: Table S8 through to Table S15.

#### Construct Validity

##### Static Balance Assessments

Outcome variables for non-instrumented static assessments included time or number of errors. Instrumented assessments reported COM displacement, and COP displacement using force plates, IMUs, smartphones, or laboratory equipment. Time since concussion ranged from 24 h to eight months, with a tendency for *insufficient* sensitivity as time increased. Assessments included the BESS (*n* = 3), instrumented BESS (*n* = 2), balance accelerometry measure (BAM) (*n* = 1), mBESS (*n* = 7), instrumented mBESS (*n* = 4), SOT (*n* = 3), balance tracking system (*n* = 1), modified clinical test of sensory interaction in balance (MCTSIB) (*n* = 1), instrumented MCTSIB (*n* = 1), Phybrata system (*n* = 1), and virtual reality static balance (*n* = 1). On average, non-instrumented assessments, BESS and mBESS displayed *sufficient* sensitivity when conducted within 48 h of sustaining a concussion [42, 43]. However, sensitivity was *insufficient* when conducted beyond this period, and up to two months post-concussion [44]. Instrumented BESS displayed *sufficient* sensitivity up to six months post-concussion [45]. Virtual reality balance and Phybrata system displayed *sufficient* sensitivity at 10- and 30-days, respectively, and are a promising alternative to current assessments if equipment is made more readily available for clinicians [46, 47].

##### Dynamic Balance Assessments

Outcome variables for non-instrumented assessments included time, heart rate, or number of errors. Instrumented assessments reported COM displacement, COP displacement, or reach distance using force plates, IMUs or laboratory equipment. Time since concussion ranged from 24 h to eight months, with a tendency for *insufficient* sensitivity as time increased. Assessments included physical and neurological examination of subtle signs (PANESS) (*n* = 1), community balance and mobility scale (*n* = 2), Kasch pulse recovery test (KPR) (*n* = 1), instrumented Y balance test (YBT) (*n* = 1), battery assessments (*n* = 2), Computer-Assisted Rehabilitation ENvironment (CAREN) system (*n* = 1). The KPR test displayed *sufficient* sensitivity when conducted on adolescents [48]. All assessments except for the battery assessments displayed *sufficient* sensitivity for adult populations. However, only the PANESS assessment reported time since concussion, with *sufficient* sensitivity up to 14-days post-concussion.

##### Gait Assessments

Outcome variables for non-instrumented gait assessments included time to complete or number of errors. Instrumented assessments provided more objective outcomes, including COM displacement, step length, step time, cadence, anterior–posterior and medio-lateral accelerations, and gait velocity using pressure sensitive walkways, IMUs, smartphones, or other laboratory equipment. Time since concussion ranged from same day to three years, with a tendency for *insufficient* sensitivity as time increased. Assessments included functional gait assessment (*n* = 2), tandem gait (*n* = 5), complex tandem gait (*n* = 1), dual-task tandem gait (*n* = 3), dual-task gait (*n* = 1), instrumented gait (*n* = 3), instrumented dual-task gait (*n* = 3), and battery of gait assessments (*n* = 1). In general, sensitivity remained *sufficient* for up to two weeks for instrumented assessments and seven days for non-instrumented assessments. Time to complete task was the primary outcome measure for non-instrumented assessments.

##### Other Assessments

Other assessments included a military-specific assessment, the Warrior Test of Tactile Agility (*n* = 1). This assessment was performed two years post-concussion and required participants to perform various motor tasks including: forward/backward run, lateral shuffle, combat roll, and changes in position (e.g. lying to standing). The lowering and rolling movements within the assessment battery demonstrated *sufficient* AUC (0.83) [49].

#### Known-Group Validity

For known-group validity, static balance included paediatric clinical test of sensory interaction in balance (PCTSIB) (*n* = 1), mBESS (*n* = 2), virtual reality balance (*n* = 1). Outcome variables were time and number of errors for non-instrumented assessments. Instrumented versions assessed COP displacement. Time since concussion averaged 7 days for all assessments. Dynamic assessments included Bruininks–Oseretsky test of motor proficiency (*n* = 1), and Postural Stress Test (PST) (*n* = 1). Outcome measures for PST assessed weight required for counterbalance. Bruininks–Oseretsky test of motor proficiency measured number of errors and time to complete. Both assessments were conducted at 1-week and 3-month time periods. Gait assessments included tandem gait (*n* = 1), dual-task tandem gait (*n* = 1), gait (*n* = 1), instrumented gait (*n* = 1), dual-task gait (*n* = 1), instrumented dual-task gait (*n* = 1). Time since concussion ranged from seven days to three years. Other assessments included the run-roll-aim task (*n* = 1) and the Portable Warrior Test of Tactile Agility (*n* = 2). Both mBESS and virtual reality static balance showed significant between-group differences when conducted within 10-days of sustaining a concussion. Both dynamic assessments displayed significant between-group differences up to three months post-concussion. However, reliance on specialised equipment reduces their feasibility for clinicians. Gait assessments include single- and dual-task tandem gait, and gait also showed significant between-group differences when conducted seven days post-concussion.

Athletes from contact and non-contact sports (*n* = 2533; 97%) were included, as well as military personnel (*n* = 83; 3%) who had been diagnosed with concussion. The most common test was the mBESS, representing 16% of all tests.

##### Measurement Error

The measurement error of 13 lower-limb motor assessments was assessed over 10 different studies. Quality ranged from adequate to very good. Assessments were categorised into static balance (*n* = 5), dynamic balance (*n* = 2), and gait (*n* = 6). Static balance assessments included BESS (*n* = 1), instrumented BESS (*n* = 1), SOT (*n* = 1), instrumented SWAY balance (*n* = 1), instrumented single leg stance (*n* = 1). Studies reported the standard error of the measure (SEM), limits of agreement (LOA), or minimal detectable change (MDC). The instrumented BESS (SEM = 0.04–0.45) and instrumented single leg stance (SEM = 0.49–2.97) displayed the lowest SEM [37, 64]. Dynamic assessments included the instrumented limits of stability test (*n* = 1) and the DPSI (*n* = 1). Both single-task (SEM = 0.0047–0.023) and dual-task (SEM = 0.004–0.019) variations displayed the lowest SEM [64]. Gait assessments included tandem gait (*n* = 2), dual-task tandem gait (*n* = 2), instrumented dual-task tandem gait (*n* = 1), instrumented gait (*n* = 4), instrumented dual-task gait (*n* = 1), and a gait battery assessment (*n* = 1). All gait assessments displayed low SEM across trials, therefore promoting the use of instrumented or non-instrumented gait assessments as acceptable tools to measure motor changes. A summary of study characteristics is presented in Table 2. Full details of the studies’ characteristics are presented in Additional file 1: Table S16 through to Table S18.

## Discussion

This systematic review aimed to [1] consolidate the reliability and validity of motor function assessments across the time course of concussion management, and [2] summarise their feasibility for clinicians and other end-users. In general, instrumented assessments providing objective analysis tended to offer superior reliability and validity compared with non-instrumented, subjective assessments, but may not be feasible for all users. Gait-based assessments showed the best reliability, with instrumented methods offering a range of outcome variables. Sensitivity is improved with an objective method of assessing performance, on more complex tasks, and during the acute stages of injury. Non-instrumented assessments offer greater practical utility, but this may be at the expense of reliability and validity, particularly beyond two weeks post-concussion. Overall, each assessment had limitations, and practitioners should be mindful of these when selecting the most appropriate assessment for their setting. However, best practice encourages practitioners to use a variety of assessments within a battery to accurately assess the multitude of symptoms experienced. Solely relying on a single-assessor, subjective diagnostic test to guide the RTP or return-to-duty process should be avoided. When selecting appropriate assessments and interpreting results, reliability, validity, and feasibility should be considered. Where possible, practitioners should use instrumented assessments for which the error, reliability and validity have been established, and a range of outcome variables can be monitored.

### Reliability

In general, objective testing from instrumented assessments offered greater test–retest reliability compared with subjective. Instrumented assessments also offer clinicians more detailed measures of motor function, thus providing a more comprehensive analysis of readiness for RTP [97].

#### Static Balance Assessments

Test–retest reliability for static assessments varied between subjective and objective measurements. In general, non-instrumented assessments relying on subjective interpretation, such as the BESS and mBESS, displayed *insufficient* reliability across multiple testing points ranging from two days to 20 months [33, 34, 50, 52, 53]. However, improved reliability was reported for both of these assessments when an increased number of trials was performed and a minimum of two assessors were present [33]. Due to a suggested learning effect associated with the BESS and mBESS, it was found that allowing a practice trial followed by 2–3 subsequent test trials produced the best reliability, taking around 10 min to administer [33]. The BESS displayed greatest test–retest reliability when more than 2 trials of 4 conditions (excluding double leg stance) were performed [34]. This differs from standard practice, where practitioners are to perform a single assessment as a means of evaluating balance deficits. Although this approach is more feasible for clinicians, only one study displayed *sufficient* reliability with one trial (*r* = 0.78) [32], with other studies showing greater reliability with multiple trials [33, 34]. Best practice would be to perform multiple trials as a single trial likely jeopardises the reliability of the assessment, limiting its justification for inclusion. Therefore, clinicians need to decide which takes priority; reliability of the measure, or practicality of its implementation. Differences in interpretation of errors between assessors also contribute to the *insufficient* reliability of these tools [98]. These differences between assessors may be exacerbated when performed on concussed individuals during the acute stage of injury due to an increased number of balance errors offering a greater capacity for disagreement to occur. Previous findings have shown that making recommendations based on the average of 3 different clinicians’ assessments and providing clear guidelines on how to administer and score the test may assist in improving reliability [98], although this may not be viable in many practical settings. Instrumented static balance assessments that offered objective outcomes displayed *sufficient* reliability, with the instrumented BESS, balance tracking system, and BAM superior to other instrumented static assessments. Of these the BAM, utilising accelerometers, may be a more feasible and cost-effective option for clinicians as opposed to using force plates. Being aware of the inherent noise and the MDC of these assessments is vital for making decisions on changes in performance. For example, the BESS has shown MDC of 7.3 errors for test–retest [37]; however, studies have shown that an average of 3–7 errors is typically performed post-concussion [13, 99]. Therefore, the test may lack the sensitivity to detect important balance deficits beyond the acute stages of injury. Instrumented static assessments (i.e. with a force plate or IMU) should be selected over non-instrumented methods wherever possible. If practitioners are working in settings that only permit non-instrumented, static assessments, they should ensure that there is *sufficient* familiarisation prior to scoring, use multiple assessors, and ensure that there are clear scoring guidelines. If these criteria cannot be met, justification for conducting the assessment beyond diagnosis should be scrutinised in future standardised assessment protocols.

#### Dynamic Balance Assessments

Few studies analysed the reliability of dynamic assessments, with results favouring the use of dynamic assessments over static. Only one study assessed the reliability of a non-instrumented dynamic motor response assessment with clinical reaction time (modified drop-stick test) [50]. While this study demonstrated *insufficient* test–retest reliability (ICC = 0.32) over an 11-month timeframe [50], reliability may be improved over shorter time periods. Instrumented dynamic assessments, on average, displayed clinically acceptable reliability (*r* = 0.32 to 0.99) when conducted within 10-days. Force plates sampling at 100–1200 Hz were shown to be useful when assessing postural sway [36, 64], but may not be readily available for all clinicians. Alternatively, IMUs also demonstrated *sufficient* reliability during the Y balance test (ICC = 0.76–0.99) [35] and may be a more feasible option for clinicians. For those who do not have access to the required equipment, non-instrumented gait assessments are recommended.

#### Gait Assessments

In general, gait assessments were seen to have the greatest test–retest reliability when compared to static and dynamic balance tests. Non-instrumented tandem gait assessments focusing on temporal gait parameters (i.e. time to complete, cadence) showed *sufficient* reliability across most studies [59, 60, 82–84]. However, test–retest reliability was *insufficient* when conducted beyond two months. This presents an issue when relying on pre-season baseline testing of tandem gait (such as during the SCAT6 protocol [100]) to interpret post-concussion scores. Therefore, if subjective assessments are to be used, it is recommended that practitioners are aware of the reliability and conduct baseline assessments in line with these timepoints.

Instrumented gait assessments assessing temporal and spatial (i.e. stride length) gait parameters also demonstrated *sufficient* reliability. Lumbar and foot-mounted IMUs were clinically acceptable and offer clinicians an inexpensive and reliable alternative to laboratory equipment [86–88]. Smartphone apps measuring movement vectors also displayed *sufficient* test–retest reliability when firmly positioned on the body [86, 88, 89, 93], but exhibited *insufficient* reliability when held in the hand. Measures of step length, step time, gait velocity, and cadence when derived from placement at the lumbar spine, or pelvis (anteriorly via belt) were most reliable [89]. The use of laboratory equipment such as 3D motion capture or a GAITRite system also displayed *sufficient* reliability across trials [89, 90]; however, the associated equipment costs and expertise requirements reduce the feasibility of these tools in most situations. Feasibility is also compromised due to the difficulty in obtaining baseline pre-injury scores, meaning normative or control comparisons are needed. Researchers should aim to develop a more readily available means of capturing pre-concussion baseline scores using commercially available technologies such as smartphones, IMU and global navigation satellite systems (GNSS) devices.

#### Considerations

Clinicians should be encouraged to implement dynamic balance or gait-based assessments as a part of a comprehensive and multifaceted concussion assessment approach, due to their higher test–retest reliability than static approaches. As previously mentioned, consistency across trials allows variations in motor strategies to be more easily detected, when a concussion is sustained [25]. Multiple trials, with the average taken, should be completed if performing non-instrumented static assessments [33], with the assessment made by multiple clinicians, in preference to one to minimise noise in the measurement and allow for smaller changes in performance to be detected as real changes [98]. Additionally, clinicians should also be mindful of time between repeated measures. Objective measures drawn from instrumented assessments provide better test–retest reliability, place less pressure on the clinician, and limit the ability of players to hide symptoms. The use of more clinically practical tools such as IMUs or smartphones, which are reliable for use in dynamic and gait-based tasks [35, 86–89], should be encouraged.

### Validity

Validity ratings of assessments ranged from *sufficient* to *insufficient* based on COSMIN guidelines [29]. In general, dynamic balance and gait assessments offered greater validity when compared with static assessments. However, validity was compromised across all assessments as time since concussion increased beyond seven days, which is likely an artefact of partial or complete recovery from the concussion beyond this point.

#### Static Balance Assessments

Construct validity for static assessments varied, with instrumented assessments offering better validity when compared with non-instrumented. The commonly used subjective assessments BESS and mBESS displayed *insufficient* ability to discriminate between groups when performed more than 48 h post-concussion, but had *sufficient* validity when performed within 24 h [42–44, 54–57]. Therefore, these assessments may aid in diagnosis; however, caution should be applied if implementing as part of a RTP protocol. Traditional models of SRC management include the assessment of subjective static balance (mBESS) to assist with decisions regarding RTP [16, 97]. Whilst instrumenting these assessments with a force plate or IMU improves sensitivity, they are still limited beyond two weeks post-injury [45, 66]. Motor function entails a complex hierarchy of integration between systems and therefore needs to be assessed along a spectrum of varying complexity [97]. During the acute stages, athletes demonstrate a significant increase in errors when performing the mBESS, but return to baseline 3–5 days post-concussion [97, 101]. Due to the gross outcome measures and suggested learning effect, it is believed that these assessments are unable to challenge the sensorimotor system to identify any underlying deficits in motor function [97]. Further, these simple static tasks are not reflective of the complex dynamic athletic tasks performed, such as running and tackling.

Virtual reality static balance using a 3D projection system displayed *sufficient* ability to discriminate between concussed and non-concussed (0.857) when conducted 10-days post-concussion [47]. This highlights the promise of the use of virtual reality technology in monitoring concussion symptoms, although the equipment is not readily available in most practical settings, thereby reducing its feasibility.

#### Dynamic Balance Assessments

In general, dynamic balance assessments displayed better construct validity than static balance assessments. However, these were still limited beyond two weeks post-concussion. Findings highlighted the importance of test selection relative to the population being assessed. In particular, the KPR test displayed *sufficient* sensitivity for children and may be a feasible option for assessing readiness for RTP in this population [48]. The PANESS, community balance and mobility scale, and instrumented Y balance test all demonstrated *sufficient* sensitivity in adult populations (0.76 – 1.00) when conducted within two weeks post-concussion [75, 76, 80, 81]. Like static assessments, these tasks are unlikely to challenge the neuromuscular system beyond the acute stage of injury. Using them to monitor changes across a graduated RTP protocol may not be best practice, particularly in concussions where symptoms persist beyond two weeks.

#### Gait Assessments

Validity of gait assessments varied amongst studies. The functional gait assessment ranged from *insufficient* to *sufficient* sensitivity (0.05–0.75) [80, 81], with higher sensitivity found when performing the assessment within one week post-concussion. Therefore, clinicians should be cautious if implementing this assessment tool beyond this time. Assessment of gait speed during normal and tandem gait, in general, demonstrated *sufficient* sensitivity when conducted within 1 week post-concussion [43, 54, 55, 58, 80, 81, 84]. Dual-task gait displayed sufficient sensitivity for children when conducted within two weeks of sustaining a SRC [85]. However, clinicians should be mindful of using gross measures of gait (e.g. time taken), due to the lack of outcome measures provided. The addition of a cognitive task (dual-task) improved sensitivity for most studies [54, 58, 84] when completed 1 week post-concussion [56]. Instrumented gait assessments had mixed results. Assessment of single- and dual-task gait using lumbar and foot-mounted IMUs amongst adult populations within five days of sustaining a concussion demonstrated *insufficient* sensitivity for gait speed, cadence, and stride length when comparing to normative reference values [92]. However, measures of single-task gait velocity and cadence using a smartphone affixed to the lumbar spine demonstrated *sufficient* AUC and between-group differences for adolescent populations with concussion when conducted one week post-injury (0.76–0.79) [58]. Like tandem gait assessments, the addition of a cognitive task improved sensitivity. Dual-task conditions aim to highlight potential deficits in attention allocation and executive function. Typically, these are observable through increased errors in a cognitive task, or variability in gait tasks [102]. Although these assessments tend to provide greater sensitivity than single-task versions, limitations still exist beyond two weeks post-concussion [95]. The use of a virtual reality system three months post-concussion displayed *sufficient* AUC (0.79–0.84) and significant between-group differences for reaction time and lateral movement asymmetries during a reactive movement task [94]. However, further research is warranted due to the small sample size used within this study. Additionally, the need for normative data currently reduces the utility of this assessment. An instrumented battery gait assessment conducted one week post-concussion, consisting of gait velocity, cadence, tandem gait time, and dual-task tandem gait time displayed *sufficient* sensitivity and specificity when all measures were combined (AUC = 0.91) [58]. However, time taken to conduct may be a barrier. Clinicians are encouraged to implement gait assessments where possible due to their ability to better classify those with and without SRC. Instrumented versions using laboratory equipment or more feasible tools such as IMUs or smartphones are the preferred option.

#### Other Assessments

The military-specific run-roll-aim assessment demonstrated statistically significant differences between concussed and control participants for ability to complete the task within two weeks post-concussion [41]. No differences were found between total time, number of correct targets identified, or delay in reaction time for cognitive stimulus, otherwise referred to as Stroop effects committed. The Portable Warrior Test of Tactile Agility demonstrated statistically significant differences in time to complete for both single-and dual-task variations [96]. The instrumental version of this assessment, utilising IMUs, demonstrated *sufficient* ability to discriminate between concussed and control during the ‘lowering and rolling’ movements (AUC = 0.83) [49]. No statistically significant differences for other portions of the assessment were seen.

#### Considerations

In general, instrumented assessments demonstrated a better ability to discriminate between concussed and non-concussed individuals. Measures of static balance were more accurate via the use of force plates [45, 61] or a 3D virtual reality projection system [47]. However, limitations surrounding suggested learning effects, and the utility of these devices, such as costs and low ecological validity, does limit their application throughout the management process following concussion. Both instrumented and non-instrumented dynamic balance assessments displayed *sufficient* sensitivity when conducted within two weeks post-concussion, therefore offering cost effective and more objective options for clinicians. Assessing time to complete on dual-task tandem gait was shown to be a sensitive and cost-effective assessment that clinicians could easily implement if access to instrumented versions is not feasible [54, 58, 84]. However, this does not provide clinicians with a variety of outcome measures, nor does it have any use beyond the acute stages of concussion [1, 103].

In general, sensitivity of assessments reduced as time from initial injury increased, which is unsurprising given the varied time course of recovery between individuals. Furthermore, sensitivity of both static [45, 73] and gait [95] assessments was reduced beyond two weeks post-concussion, meaning clinicians must be cautious when using these assessments as a RTP measure beyond this timeframe. Athletes returning to play following a concussion have shown an increased risk of acute musculoskeletal injury [21, 104, 105]. It is suggested that subclinical neuromuscular deficits may linger beyond expected recovery timeframes, but due to poor assessment availability and limited research surrounding best-care concussion management, many of these changes go undetected [21, 97, 104, 105]. This review provides clinicians with reliability and validity measures of assessments to allow a more educated selection of tests. However, it also highlights the problems with concussion management protocols, specifically the over-reliance on tools not initially designed to inform RTP decisions.

### Feasibility and Utility

This review aimed to summarise the reliability and validity of lower-limb motor assessments for the management of SRC. However, what should not be overlooked is the clinical utility and feasibility of such assessments and their seamless integration within a RTP or return-to-duty protocol. Aside from the reliability and validity of a measure, stakeholders must also consider other factors such as interpretability of outcomes, cost of equipment, expertise required, and time needed for implementation and analysis of results, when developing assessment protocols. In general, instrumented assessments demonstrated better test–retest reliability across multiple time periods as well as better ability to discriminate between concussed and non-concussed individuals. Of these, laboratory assessments using force plates, 3D motion capture, or pressure-sensitive walkways provided clinicians with more accurate objective measures. However, these display low ecological validity for the assessment of field-based sports due to the controlled environmental conditions [103] and lack of flexibility in tasks that can be performed and therefore may have poor crossover to the stochastic nature of sports competition. Equipment and facility requirements are typically associated with high cost and therefore not feasible for most team-sports [103]. Furthermore, the need for trained personnel to collect and analyse the data may act as further barriers to their uptake within practice.

Other tools used for instrumented assessments included IMUs and smartphone devices. These tools were shown to have better test–retest reliability and validity for most assessment categories (static, dynamic, gait). Studies included in this review assessed the validity and reliability of lumbar and foot-mounted IMUs [35, 86–88]. Test–retest reliability for dynamic and gait assessments using these devices were similar to those from laboratory assessments. Similar findings were associated with the use of smartphone devices, displaying *sufficient* test–retest reliability during gait assessments [71, 89]. Although they achieved poorer validity than laboratory equipment, IMUs and smartphone devices offered clinically acceptable validity, specifically during dynamic balance and gait assessments [58, 76, 79, 92, 95]. In regard to interpretability of results, cadence and gait velocity metrics derived from IMUs and smartphones displayed sufficient ability to discriminate between concussed and non-concussed. Typically, these measures are made readily available for clinicians when using the appropriate software for the respective device and therefore avoid the need for additional analysis. As such, the lower cost, autonomy for analysis, and greater portability of these devices may improve their uptake in the field. These devices may offer practitioners the ability to identify at-risk individuals who require further investigation through more in-depth assessments. Efforts should be made to make these instrumented assessments more feasible for end-users without compromising reliability or validity. Utilising technologies such as IMUs embedded in current wearable technologies (e.g. GPS units, smartphones and watches) should be explored further.

## Conclusions

Based on the findings from this review, clinicians are encouraged to implement instrumented or non-instrumented dynamic balance or gait assessments as part of a battery of assessments and not in isolation. Instrumented assessments utilising more complex gait tasks should be encouraged to add resolution to existing RTP protocols. On average, static assessments displayed *insufficient* test–retest reliability and validity for the management of SRC. If practitioners do not have the resources to perform instrumented tests, it is recommended that they consider the reliability and validity issues that potentially limit the simpler test options. Future research should aim to establish standardised protocols and best practice for monitoring motor function during the RTP period and beyond. Developing the use of accessible technologies such as IMUs, smartphones and the use of marker-less tracking to monitor gait function is an important step for concussion management. Furthermore, understanding how movement changes under more context-specific scenarios, where fatigue, decision-making, and the performance of more complex movements occur, is warranted.

### Supplementary Information

**Additional file 1.** Supplementary Tables 1–18.

## Data Availability

Data sharing is not applicable to this article as no datasets were generated or analysed during the current study.

## Abbreviations

mTBI: Mild traumatic brain injury
SRC: Sports-related concussion
RTP: Return to play
BESS: Balance error scoring system
mBESS: Modified balance error scoring system
SOT: Sensory organisation test
PRISMA: Preferred Reporting Items for Systematic Reviews and Meta-analyses
COSMIN: COnsensus-based Standards for the selection of health Measurement INstruments
ClinROMs: Clinical reported outcome measures
BAM: Balance accelerometry measure
TUG: Timed up and go
MCTSIB: Modified clinical test of sensory interaction in balance
PCTSIB: Paediatric clinical test of sensory interaction in balance
PANESS: Physical and neurological examination of subtle signs
KPR: Kasch pulse recovery
CAREN system: Computer-assisted rehabilitation environment system
SLS: Single leg stance
LOS: Limits of stability
DPSI: Dynamic postural stability index
PST: Postural stress test
BESTest: Balance evaluations system test
YBT: Y-balance test
DT: Dual-task
Sens: Sensitivity
Spec: Specificity
AUC: Area under the curve
COP: Centre of pressure
COM: Centre of mass
SEM: Systematic error of the measure
MDC: Minimal detectable change
IMU: Inertial measurement unit
GNSS: Global navigation satellite system
