# Supplementary material for: Validity and Reliability of Methods to Assess Movement Deficiencies Following Concussion: A COSMIN Systematic Review
Source: Sports Med Open. 2023 Aug 14;9:76. doi: 10.1186/s40798-023-00625-0 (PMC10425315; doi:10.1186/s40798-023-00625-0)
Supplement: Supplementary file 1 — Additional file 1. Supplementary Tables 1–18. [file 40798_2023_625_MOESM1_ESM.docx]

# Supplementary material

# Validity and Reliability of Methods to Assess Movement Deficiencies Following Concussion: A COSMIN Systematic Review

Laura A M Dunne^a,b^, Michael H Cole^a,c^, Stuart J Cormack^b,d^, David R Howell^e,f^, and Rich D Johnston^a,b,g^

^a^ School of Behavioural and Health Sciences, Australian Catholic University, Brisbane, Australia

^b^ SPRINT Research Centre, Faculty of Health Sciences, Australian Catholic University, Australia

^c^ Healthy Brain and Mind Research Centre, Faculty of Health Sciences, Australian Catholic University, Australia

^d^ School of Behavioural and Health Sciences, Australian Catholic University, Melbourne, Australia

^e^ Sports Medicine Center, Children's Hospital Colorado, Aurora, Colorado USA;

^f^ Department of Orthopedics, University of Colorado School of Medicine, Aurora, Colorado, USA

^g^ Carnegie Applied Rugby Research Centre, School of Sport, Leeds Beckett University, United Kingdom

| Table S1. Test-retest of static balance assessments | | | | | | |
| --- | --- | --- | --- | --- | --- | --- |
|  | Participants | Age | Time Interval | Method Quality | Results | Measurement Quality Rating |
| *Balance Error Scoring System* | | | | | | |
| Broglio et al., 2009 | n = 48 | ~20 years | ~50 days | Adequate | 2 trials: G = 0.82-0.85 | + |
| Hunt et al., 2009 | n = 78 | ~16 years | NA | Adequate | 1-7 trials: r = 0.60-0.94 | -/+ |
| Chang et al., 2014 | n = 30 | ~24 years | 7 days | Adequate | r = 0.61-0.78 | + |
| Broglio et al., 2018 | n = 4874 | ~19 years | ~11 months  ~ 20 months | Adequate | r = 0.41  r = 0.42 | -  - |
| Finnoff et al., 2009 | n = 30 |  | 2 days | Adequate | r = 0.57 | - |
| Alsalaheen et al., 2015 | n = 36 | ~15 years | 7 days | Adequate | r = 0.13-0.74 | -/+ |
| *Instrumented* *Balance Error Scoring System* | | | | | | |
| Chang et al., 2014 | n = 30 | ~24 years | 7 days | Adequate | Force plate: r = 0.89  WBB: r = 0.88 | +  + |
| Alsalaheen et al., 2015 | n = 36 | ~15 years | 7 days | Adequate | r = 0.19-0.74 | -/+ |
| *Modified* *Balance Error Scoring System* | | | | | | |
| Hunt et al., 2009 | n = 144 | ~16 years | NA | Adequate | 3 trials: r = 0.73-0.88 | + |
| Nelson et al., 2017 | n = 57 | 9-13 years | ~2 months | Adequate | 2 trials: r = 0.02 | - |
| Kontos et al., 2021 | n = 108 | 18-40 years | ~ 6 months | Adequate | r = 0.59 | - |
| *Instrumented Modified Balance Error Scoring System* | | | | | | |
| German et al., 2022 | n = 28 | ~28 years | Same day | Adequate | Force plate: r = 0.69-0.77  Phone: r = 0.66-0.75 | -/+  -/+ |
| *Single Leg Stance* | | | | | | |
| Schneiders et al., 2008 | n = 30 | ~21 years | 15 mins | Doubtful | r = 0.85 | + |
| Schneiders et al., 2010 | n = 172 | ~22 years | ~1 week | Adequate | r = 0.85 | + |
| *Instrumented single leg stance* | | | | | | |
| Westwood et al., 2020 | n = 12 | ~22 years | 10 days | Adequate | Eyes Open  r = 0.58-0.84  Eyes Closed  r = 0.77-0.88  ML r = 0.77  AP r = 0.87  Vertical r = 0.88 | -  +  + |
| *Balance Accelerometry Measure* | | | | | | |
| Marchetti et al., 2013 | n = 84 | ~47 years | 15 mins | Adequate | r = 0.74-0.86 | + |
| Salisbury et al., 2018 | n = 42 | ~24 years | Same day | Doubtful | r = 0.28-0.82 | -/+ |
| *Balance tracking system* | | | | | | |
| Hearn et al., 2018 | n = 20 | ~23 years | 1, 3, 8, 15 days | Adequate | r = 0.92 | + |
| *SWAY Balance mobile application* | | | | | | |
| Amick et al., 2015 | n = 24 | ~26 years | 1 week  2 weeks  3 weeks | Adequate | r = 0.47  r = 0.78  r = 0.75 | -  +  + |
| *Sensory Organisation Test* | | | | | | |
| Register et al., 2013 | n = 38 | ~21 years | ~1 month | Adequate | r = 0.3 | - |
| Christy et al., 2019 | n = 87 | ~21 years | 7-12 months | Adequate | r = 0.72 | + |

| Table S2. Inter-tester reliability of static balance assessments. | | | | | | |
| --- | --- | --- | --- | --- | --- | --- |
|  | Participants | Age | No of Assessors | Method Quality | Results | Measurement Quality Rating |
| *Balance Error Scoring System* | | | | | | |
| Finnoff et al., 2009 | n = 30 | >18 years | n = 3 | Adequate | r = 0.57 | - |
| Chang et al., 2014 | n = 30 | ~24 years | n = 3 | Adequate | Total r = 0.20-0.35  Individual r = 0.00-0.69 | -  - |
| Riemann et al., 1999 | n = 111 | ~20 years | n = 18 | Adequate | Stance variations: r = 0.65-0.96 | -/+ |
| Kleffelgaard et al., 2018 | n = 42 | ~23 years | n = 2 | Adequate | r = 0.66 | - |
| *Modified Balance Error Scoring System* | | | | | | |
| Kleffelgaard et al., 2018 | n = 42 | ~23 years | n = 2 | Adequate | r = 0.80 | + |
| Glass et al., 2019 | n = 25 | ~25 years | n = 3 | Adequate | r = 0.83 | + |

| Table S3. Test-retest reliability of dynamic balance assessments. | | | | | | |
| --- | --- | --- | --- | --- | --- | --- |
|  | Participants | Age | Time Interval | Method Quality | Results | Measurement Quality Rating |
| *Instrumented Y Balance Test* | | | | | | |
| Johnston et al., 2017 | n = 15 | ~23 years | Same day | Adequate | r = 0.76-0.99 | + |
| *Clinical Reaction Time* | | | | | | |
| Broglio et al., 2018 | n = 4874 | ~20 years | ~11 months | Adequate | r = 0.32 | - |
| *Instrumented Limits of Stability Test* | | | | | | |
| Lininger et al., 2018 | n = 27 | ~24 years | ~7 days | Adequate | r = 0.95-0.96 | + |
| Alsalaheen et al., 2015 | n = 15 | ~16 years | 7 days | Adequate | r = 0.73-0.96 | + |
| *Dynamic Postural Stability Index (DPSI) and DPSI with Dual Task* | | | | | | |
| Westwood et al., 2020 | n = 12 | ~22 years | 10 days | Adequate | DPSI r = 0.58-0.93  DPSI DT r = 0.32-0.80 | -/+  -/+ |

| Table S4. Test-retest reliability of gait-based motor assessments. | | | | | | |
| --- | --- | --- | --- | --- | --- | --- |
|  | Participants | Age | Time Interval | Method Quality | Results | Measurement Quality Rating |
| *Tandem Gait* | | | | | | |
| Schneiders et al., 2008 | n = 30 | ~20 years | Same day | Doubtful | r = 0.98 | + |
| Schneiders et al., 2010 | n = 172 | ~22 years | ~7 days | Adequate | r = 0.97 | + |
| Eemanipure et al., 2012 | n = 133 | ~25 years | 7 days | Adequate | r = 0.95 | + |
| Nelson et al., 2017 | n = 155 | 5-13 years | ~2-4 months | Adequate | r = 0.46 | - |
| Howell et al., 2019 | n = 32 | ~14 years | 2, 4 weeks | Adequate | r = 0.86 | + |
| Wingerson et al., 2020 | n = 44 | ~15 years | ~7 days | Doubtful | r = 0.93 | + |
| *Instrumented Gait* | | | | | | |
| Howell et al., 2020 | n = 20 | ~22 years | ~2 weeks | Adequate | r = 0.86-0.95 | + |
| Howell et al., 2017 | n = 265 | ~20 years | ~8 months | Adequate | r = 0.68-0.80 | -/+ |
| Nishinguchi et al., 2012 | n = 30 | ~20 years | Same day | Adequate | r = 0.75-0.91 | + |
| Kuznetsov et al., 2018 | n = 32 | ~25 years | ~7 days | Adequate | r = 0.59-0.96 | -/+ |
| Silsupadol et al., 2017 | n = 12  n = 22 | ~22 years  ~74 years | Same day | Doubtful | r = 0.70-0.99 | + |
| Howell et al., 2021 | n = 17 | ~17 years | ~1 month | Adequate | r = 0.10-0.94 | -/+ |
| Wilkerson et al., 2021 | n = 9 | ~25 years | ~3 months | Adequate | r = 0.83-0.97 | + |
| *Dual Task Tandem Gait* | | | | | | |
| Howell et al., 2019 | n = 32 | ~15 years | 2, 4 weeks | Adequate | r = 0.84 | + |
| Wingerson et al., 2020 | n = 44 | ~15 years | ~7 days | Doubtful | r = 0.92 | + |
| *Instrumented Dual Task Tandem Gait* | | | | | | |
| Howell et al., 2020 | n = 20 | ~22 years | ~2 weeks | Adequate | r = 0.80-0.94 | + |
| Howell et al., 2017 | n = 265 | ~20 years | ~8 months | Adequate | r = 0.73-0.85 | + |
| *Instrumented Dual Task Gait* | | | | | | |
| Howell et al., 2021 | n = 17 | ~17 years | ~1 month | Adequate | r = 0.61-0.94 | -/+ |
| Wilkerson et al., 2021 | n = 9 | ~25 years | ~3 months | Adequate | r = 0.81-0.97 | + |
| *Timed Up and Go* | | | | | | |
| Eemanipure et al., 2012 | n = 133 | ~25 years | 7 days | Adequate | r = 0.85 | + |
| *Walking on Balance Beam* | | | | | | |
| Eemanipure et al., 2012 | n = 133 | ~25 years | 7 days | Adequate | r = 0.87 | + |

| Table S5. Inter-tester reliability of gait-based motor assessments. | | | | | | |
| --- | --- | --- | --- | --- | --- | --- |
|  | Participants | Age | No of Assessors | Method Quality | Results | Measurement Quality Rating |
| *Tandem Gait* | | | | | | |
| Eemanipure et al., 2012 | n = 133 | ~25 years | n = 2 | Adequate | r = 0.70 | + |
| *Timed Up and Go* | | | | | | |
| Eemanipure et al., 2012 | n = 133 | ~25 years | n = 2 | Adequate | r = 0.99 | + |
| *Walking on Balance Beam* | | | | | | |
| Eemanipure et al., 2012 | n = 133 | ~25 years | n = 2 | Adequate | r = 0.35 | - |

| Table S6. Internal consistency of gait-based motor assessments. | | | | | | |
| --- | --- | --- | --- | --- | --- | --- |
|  | Participants | Age | Time Interval | Method Quality | Results | Measurement Quality Rating |
| *Instrumented Gait* | | | | | | |
| Howell et al., 2016 | n = 24  n = 21 | ~15 years  ~22 years | 1 week  2 weeks  1 month  2 months | Inadequate | r = 0.76-0.97 | + |
| *Instrumented Dual Task Gait* | | | | | | |
| Howell et al., 2016 | n = 24  n = 21 | ~15 years  ~22 years | 1 week  2 weeks  1 month  2 months | Inadequate | r = 0.79-0.97 | + |

| Table S7. Inter-tester reliability of task-specific assessments. | | | | | | |
| --- | --- | --- | --- | --- | --- | --- |
|  | Participants | Age | No of Assessors | Method Quality | Results | Measurement Quality Rating |
| *Run-Roll-Aim* | | | | | | |
| Prim et al., 2019 | n = 50 | ~33 years | n = 2 | Adequate | r = 0.28-0.89 | -/+ |

| Table S8. Discriminant validity of static balance assessments. | | | | | | |
| --- | --- | --- | --- | --- | --- | --- |
|  | Participants | Age | Time since concussion | Method Quality | Results | Measurement Quality Rating |
| *Balance Error Scoring System* | | | | | | |
| Buckley et al., 2018 | n = 35 | ~18 years | <24 hours | Adequate | Sensitivity = 0.60 | - |
| Oldham et al., 2018 | n = 38  n = 38 control | ~20 years | <48 hours | Very Good | Sensitivity = 0.45  Specificity = 0.50  AUC = 0.51 | -  -  - |
| King et al., 2014 | n = 13  n = 13 control | ~16 years | 2-5 months | Very Good | Sensitivity = 0.23  Specificity = 0.92  AUC = 0.63 | -  +  - |
| *Instrumented Balance Error Scoring System* | | | | | | |
| King et al., 2014 | n = 13  n = 13 control | ~16 years |  | Very Good | Sensitivity = 0.38  Specificity = 1  AUC = 0.70 | -  +  + |
| Pryhoda et al., 2020 | n = 25  n = 92 control | >18 years | 3 days  1 week  1 month  6 months | Adequate | r = 0.69-0.94  r = 0.53-0.84  r = 0.41-0.92  r = 0.60-0.80 | -/+  -/+  -/+  -/+ |
| *Balance Accelerometry Measure (BAM)* | | | | | | |
| Furman et al., 2013 | n = 43  n = 27 control | ~16 years | <2 weeks  >2weeks | Very Good | AUC = 0.74-0.77  AUC = 0.67-0.68 | +  - |
| *Modified Balance Error Scoring System* | | | | | | |
| VanDeventer et al., 2021 | n = 81  n = 58 control | ~15 years |  | Very Good | AUC = 0.71 | + |
| Hanninen et al., 2018 | n = 27  n = 179 control | ~28 years | Day of injury | Adequate | AUC = 0.69-0.72 | -/+ |
| Corwin et al., 2020 | n = 78  n = 88 control | ~16 years | ~2 weeks | Very Good | Sensitivity = 0.05-0.60 | - |
| Buckley et al., 2018 | n = 35 | ~18 years | <24 hours  RTP | Adequate | Sensitivity = 0.71  Sensitivity = 0.65 | +  - |
| Oldham et al., 2018 | n = 38  n = 38 control | ~20 years | <48 hours | Very Good | Sensitivity = 0.45  Specificity = 0.63  AUC = 0.54 | -  -  - |
| King et al., 2017 | n = 52  n = 76 control | ~20 years | ~3 days | Very Good | Sensitivity = 0.35  AUC = 0.61 | -  - |
| King et al., 2014 | n = 13  n = 13 control | ~16 years | ~ | Very Good | Sensitivity = 0.31  Specificity = 0.85  AUC = 0.64 | -  +  - |
| *Instrumented Modified Balance Error Scoring System* | | | | | | |
| King et al., 2017 | n = 52  n = 76 | ~20 years | ~3 days | Very Good | Sensitivity = 0.59-0.63  AUC = 0.74-0.75 | -  + |
| Doherty et al., 2017 | n = 15  n = 15 control | ~22 years |  | Very Good | Sensitivity = 0.72-0.92  Specificity = 0.79-0.96  AUC = 0.91 | +  +  + |
| King et al., 2014 | n = 13  n = 13 control | ~16 years | ~2-5 months | Very Good | Sensitivity = 0.54  Specificity = 1  AUC = 0.81 | -  +  + |
| Baracks et al., 2018 | n = 48  n = 45 control | ~20 years | ~ 3 days | Very Good | Sensitivity = 0.42-0.52  Specificity = 0.76-0.80 | -  + |
| *Sensory Organisation Test* | | | | | | |
| Register et al., 2013 | n = 132  n = 38 control | ~18 years | ~2 days | Very Good | Sensitivity = 0.13  Specificity = 0.95 | -  + |
| Resch et al., 2016 | n = 40  n = 40 control | ~19 years | 24 hours | Adequate | Sensitivity = 0.73  Specificity = 0.85  AUC = 0.79 | +  +  + |
| Broglio et al., 2008 | n = 63  n = 66 control | ~20 years | 24 hours  8 months | Very Good | Sensitivity = 0.29 | - |
| *Balance Tracking System* | | | | | | |
| Goble et al., 2016 | n = 25 | ~20 years | 48 hours | Adequate | Sensitivity = 0.64 | - |
| *Instrumented Modified Clinical Test of Sensory Interaction in Balance (MCTSIB)* | | | | | | |
| Toong et al., 2021 | n = 32  n = 32 control | ~14 years | ~3 months | Very Good | Sensitivity = 0.03  Specificity = 0.93 | -  + |
| *Modified Clinical Test of Sensory Interaction in Balance (MCTSIB)* | | | | | | |
| Corwin et al., 2020 | n = 78  n = 88 control | ~16 years | ~2 weeks | Very Good | Sensitivity = 0.37  Specificity = 0.88 | -  + |
| *Phybrata System (Instrumented)* | | | | | | |
| Ralston et al., 2020 | n = 92  n = 83 control | ~24 years | ~30 days | Very Good | Sensitivity = 0.92  Specificity = 0.94  AUC = 0.98 | +  +  + |
| *Virtual Reality Balance* | | | | | | |
| Teel et al., 2016 | n = 27  n = 94 control | ~20 years | ~7-10 days | Doubtful | Sensitivity = 0.86  Specificity = 0.88  AUC = 0.86 | +  +  + |

| Table S9. Discriminant validity of dynamic balance assessments | | | | | | |
| --- | --- | --- | --- | --- | --- | --- |
|  | Participants | Age | Time since concussion | Method Quality | Results | Measurement Quality Rating |
| *Physical and Neurological Examination of Subtle Signs (PANESS)* | | | | | | |
| Stephens et al., 2020 | n = 20  n = 17 control | ~15 years | ~4-14 days | Adequate | Sensitivity = 0.76  Specificity = 0.90 | +  + |
| *Community Balance and Mobility Scale* | | | | | | |
| Pape et al., 2016 | n = 8  n = 8 control | ~31 years | ~7 days | Adequate | Sensitivity = 1.00  Specificity = 0.88  AUC = 0.98 | +  +  + |
| Pape et al., 2020 | n = 45  n = 45 control | ~33 years |  | Adequate | Sensitivity = 0.78  Specificity = 0.91  AUC = 0.92 | +  +  + |
| *Kasch Pulse Recovery (KPR)* | | | | | | |
| Fyffe et al., 2020 | n = 45 | ~13 years |  | Very Good | Sensitivity = 1.00  Specificity = 0.96  AUC = 0.98 | +  +  + |
| *Instrumented Y Balance Test* | | | | | | |
| Johnston et al., 2019 | n = 21  n = 88 control | ~22 years |  | Adequate | Sensitivity = 0.76  Specificity = 0.53 | +  - |
| *Battery Assessments* | | | | | | |
| Toong et al., 2021 | n = 32  n = 32 control | ~13 years | ~1 month | Very Good | Sensitivity = 0.41  Specificity = 0.77 | -  + |
| Rao et al., 2020 | n = 11  n = 10 control | ~39 years |  | Adequate | Score of ‘no impairment’ = 89% MTBI | - |
| *CAREN System* | | | | | | |
| Rao et al., 2020 | n = 11  n = 10 control | ~39 years |  | Adequate | Sensitivity = 0.65-0.90 | -/+ |

| Table S10. Discriminant validity of gait-based motor assessments. | | | | | | |
| --- | --- | --- | --- | --- | --- | --- |
|  | Participants | Age | Time since concussion | Method Quality | Results | Measurement Quality Rating |
| *Functional Gait Assessment and Gait Speed* | | | | | | |
| Pape et al., 2020 | n = 45  n = 45 control | ~33 years |  | Adequate | Sensitivity = 0.05-0.69  Specificity = 0.76-1.00 | -/+  + |
| Pape et al., 2016 | n = 8  n = 8 control | ~31 years | ~7 days | Adequate | Sensitivity = 0.75-0.88  Specificity = 0.75 | +  + |
| *Tandem Gait* | | | | | | |
| Hanninen et al., 2018 | n = 27  n = 127 control | ~27 years | Day of injury | Adequate | AUC = 0.55-0.56 | - |
| Oldham et al., 2018 | n = 38  n = 38 control | ~20 years | <48 hours | Very Good | Sensitivity = 0.63  Specificity = 0.61  AUC = 0.70 | -  -  + |
| VanDeventer et al., 2021 | n = 81  n = 58 control | ~14 years |  | Very Good | Sensitivity = 0.88  Specificity = 0.72  AUC = 0.86 | +  +  + |
| Wingerson et al., 2020 | n = 73  n = 44 control | ~15 years | ~7 days | Very Good | AUC = 0.85 | + |
| Howell et al., 2019 | n = 15  n = 31 control | ~16 years | ~7 days | Very Good | AUC = 0.86 | + |
| *Complex Tandem Gait* | | | | | | |
| Corwin et al., 2020 | n = 78  n = 88 control | ~16 years | ~2 weeks | Very Good | Sensitivity = 0.41  Specificity = 0.90 | -  + |
| *Dual Task Tandem Gait* | | | | | | |
| VanDeventer et al., 2021 | n = 81  n = 58 control | ~14 years |  | Very Good | Sensitivity = 0.85  Specificity = 0.72  AUC = 0.84 | +  +  + |
| Wingerson et al., 2020 | n = 73  n = 44 control | ~15 years | ~7 days | Very Good | AUC = 0.80 | + |
| Howell et al., 2019 | n = 15  n = 31 control | ~16 years | ~7 days | Very Good | AUC = 0.87 | + |
| *Instrumented Gait* | | | | | | |
| Howell et al., 2019 | n = 15  n = 31 control | ~16 years | ~7 days | Very Good | AUC = 0.76-0.79 | + |
| Howell et al., 2019 | n = 54  n = 60 control | ~20 years | ~5 days | Adequate | Sensitivity = 0.26-0.63  Specificity = 0.62-0.85 | -  -/+ |
| *Dual Task Gait* | | | | | | |
| Barnes et al., 2023 | n = 27  n = 21 control | 12-18 years | ~2 weeks | Very Good | Sensitivity = 0.77  Specificity = 1.00 | + |
| *Instrumented Dual Task Gait* | | | | | | |
| Howell et al., 2015 | n = 10  n = 7 control | ~19 years | 72 hours  1 week  2 weeks  1 month  2 months | Very Good | Sensitivity = 0.70  Sensitivity = 0.70  Sensitivity = 0.60  Sensitivity = 0.30  Sensitivity = 0.40 | +  +  -  -  - |
| Howell et al., 2019 | n = 54  n = 60 control | ~20 years | ~5 days | Adequate | Sensitivity =0.26-0.56 | - |
| Wilkerson et al., 2021 | n = 9  n = 7 control | ~25 years  ~20 years | ~3 ± 2.2 years | Very Good | Reaction time dispersion (ST and DT combined)  Sensitivity = 0.89  Specificity = 0.86 | + |
| *Battery Gait Assessment* | | | | | | |
| Howell et al., 2019 | n = 15  n = 31 control | ~16 years | ~7 days | Very Good | AUC = 0.91 | + |

| Table S11. Discriminant validity of task-specific motor assessments. | | | | | | |
| --- | --- | --- | --- | --- | --- | --- |
|  | Participants | Age | Time since concussion | Method Quality | Results | Measurement Quality Rating |
| *Instrumented Portable Warrior Test of Tactile Agility* | | | | | | |
| Favorov er al., 2021 | n = 42  n = 57 control | ~29 years  ~29 years | ≤ 2 years | Very Good | Lowering and rolling movements:  AUC = 0.83 | + |

| Table S13. Know groups validity of dynamic balance assessments. | | | | | | |
| --- | --- | --- | --- | --- | --- | --- |
|  | Participants | Age | Time since concussion | Method Quality | Results | Measurement Quality Rating |
| *Bruininks-Oseretsky Test of Motor Proficiency* | | | | | | |
| Gagon et al., 2004 | n = 40  n = 40 control | ~12 years | 1 week  4 weeks  3 months | Adequate | p = 0.001  p = 0.002-0.001  p = 0.024-0.02 | +  +  + |
| *Postural Stress Test (PST)* | | | | | | |
| Gagon et al., 2004 | n = 40  n = 40 control | ~12 years | 1 week  3 months | Adequate | p = 0.027  p = 0.024 | +  + |

| Table S12. Known groups validity of static balance assessments. | | | | | | |
| --- | --- | --- | --- | --- | --- | --- |
|  | Participants | Age | Time since concussion | Method Quality | Results | Measurement Quality Rating |
| *Paediatric Clinical Test of Sensory Interaction in Balance* | | | | | | |
| Gagon et al., 2004 | n = 40  n = 40 control | ~12 years | 1 week  4 weeks  3 months | Adequate | p = 0.46-0.49 | - |
| *Modified Balance Error Scoring System* | | | | | | |
| Howell et al., 2019 | n = 15  n = 31 control | ~16 years | ~7 days | Very Good | p = 0.23-0.06 | - |
| VanDeventer et al., 2021 | n = 81  n = 58 control | ~14 years | ~6 days | Very Good | p = 0.019-<0.001 | + |
| *Virtual Reality Balance* | | | | | | |
| Teel et al., 2015 | n = 28  n = 94 control | >18 years | ~7-10 days | Doubtful | p = 0.006-<0.001 | + |

| Table S14. Known groups validity of gait-based assessments. | | | | | | |
| --- | --- | --- | --- | --- | --- | --- |
|  | Participants | Age | Time since concussion | Method Quality | Results | Measurement Quality Rating |
| *Tandem Gait* | | | | | | |
| VanDeventer et al., 2021 | n = 81  n = 58 control | ~14 years |  | Very Good | p = <0.001 | + |
| *Dual Task Tandem Gait* | | | | | | |
| VanDeventer et al., 2021 | n = 81  n = 58 control | ~14 years |  | Very Good | p = 0.002-<0.001 | + |
| *Gait* | | | | | | |
| Howell et al., 2019 | n = 15  n = 31 control | ~16 years | ~7 days | Very Good | p = 0.006 | + |
| *Instrumented Gait* | | | | | | |
| Howell et al., 2019 | n = 15  n = 31 control | ~16 years | ~7 days | Very Good | p = 0.46-0.002 | -/+ |
| Wilkerson et al., 2021 | n = 9  n = 7 control | ~25 years  ~20 years | ~3 ± 2.2 years | Adequate | Lateral: p = 0.76-0.003  Diagonal: p = 0.96-0.46 | -/+  - |
| *Dual Task Gait* | | | | | | |
| Howell et al., 2019 | n = 15  n = 31 control | ~16 years | ~7 days | Very Good | p = 0.29 | - |
| *Instrumented Dual Task Gait* | | | | | | |
| Howell et al., 2019 | n = 15  n = 31 control | ~16 years | ~7 days | Very Good | p = 0.68-0.07 | - |

| Table S15. Known groups validity of task-specific motor assessments. | | | | | | |
| --- | --- | --- | --- | --- | --- | --- |
|  | Participants | Age | Time since concussion | Method Quality | Results | Measurement Quality Rating |
| *Run-Roll-Aim* | | | | | | |
| Prim et al., 2019 | n = 33  n = 50 control | ~26 years | ~2 weeks | Very Good | p = <0.01 | + |
| *The Portable Warrior Test of Tactile Agility* | | | | | | |
| Cecchini et al., 2021 | n = 64  n = 59 control | ~29 years  ~29 years | ~5 months | Very Good | Time to complete  Single-task: p = <0.001  Dual-task: p = 0.05 | + |
| *Instrumented Portable Warrior Test of Tactile Agility* | | | | | | |
| Favorov et al., 2021 | n = 42  n = 57 control | ~29 years  ~29 years | ≤ 2 years | Very Good | Lowering portion:  p = <0.0001 | + |

| Table S16. Measurement error of static balance assessments. | | | | | | |
| --- | --- | --- | --- | --- | --- | --- |
|  | Participants | Age | Time Interval | Method Quality | Results | Measurement Quality Rating |
| *Balance Error Scoring System* | | | | | | |
| Finnoff et al., 2009 | n = 3 | >18 years |  | Adequate | MDC test-retest = 7.3  MDC inter-tester = 9.4 | ? |
| *Instrumented Balance Error Scoring System* | | | | | | |
| Riemann et al., 1999 | n = 111 | ~20 years |  | Adequate | SEM = 0.00-0.45 | ? |
| *Sensory Organisation Test* | | | | | | |
| Register et al., 2013 | n = 38 | ~20 years |  | Very Good | SEM = 4.92-5.00 | ? |
| *Instrumented SWAY Balance* | | | | | | |
| Amick et al., 2015 | n = 24 | ~25 years | 1 week  2 weeks  3 weeks | Adequate | SEM = 7.56  SEM = 5.82  SEM = 5.77 | ?  ? ? |
| *Instrumented Single Leg Stance* | | | | | | |
| Westwood et al., 2020 | n = 12 | ~22 years | ~10 days | Adequate | SEM = 0.41-2.97 | ? |

| Table S17. Measurement error of dynamic balance assessments. | | | | | | |
| --- | --- | --- | --- | --- | --- | --- |
|  | Participants | Age | Time Interval | Method Quality | Results | Measurement Quality Rating |
| *Instrumented Limits of Stability Test* | | | | | | |
| Lininger et al., 2018 | n = 27 | ~24 years | ~7 days | Very Good | SEM = 0.35-1.17 | ? |
| *Dynamic Postural Stability Index (DPSI) and DPSI with Dual Task* | | | | | | |
| Westwood et al., 2020 | n = 12 | ~22 years | ~10 days | Adequate | DPSI SEM = 0.0047-0.023  DPSI+DT SEM = 0.004-0.019 | ? |

| Table S18. Measurement error of gait-based motor assessments. | | | | | | |
| --- | --- | --- | --- | --- | --- | --- |
|  | Participants | Age | Time Interval | Method Quality | Results | Measurement Quality Rating |
| *Tandem Gait* | | | | | | |
| Howell et al., 2019 | n = 32 | ~14 years |  | Adequate | RCI = 5.3 sec | ? |
| Howell et al., 2019 | n = 15 | ~16 years | ~7 days | Very Good | SEM = 0.07 | ? |
| *Dual Task Tandem Gait* | | | | | | |
| Howell et al., 2019 | n = 32 | ~14 years |  | Adequate | RCI = 8.5 sec | ? |
| Howell et al., 2019 | n = 15 | ~16 years | ~7 days | Very Good | SEM = 0.06 | ? |
| *Instrumented Dual Task Tandem Gait* | | | | | | |
| Howell et al., 2020 | n = 20 | ~22 years | ~2 weeks | Adequate | SEM = -0.08-1.35 | ? |
| *Instrumented Gait* | | | | | | |
| Howell et al., 2016 | n = 45 | ~15-22 years | Initial  1 week  2 weeks  1 month  2 months | Very Good | MDC = 0.88-7.42 | ? |
| Kuznetsov et al., 2018 | n = 32 | ~25 years | ~7 days | Very Good | Temporal metrics SEM = 0.02-0.34  Spatial metrics SEM = 0.32-4.10 | ? |
| Howell et al., 2020 | n = 20 | ~22 years | ~2 weeks | Adequate | Discrete trials SE =  -0.50-0.04  2 min trial SE = -1.32—0.08 | ? |
| Howell et al., 2019 | n = 31 | ~16 years | ~7 days | Very Good | SEM = 0.07-0.08 | ? |
| *Instrumented Dual Task Gait* | | | | | | |
| Howell et al., 2016 | n = 45 | ~15-22 years | Initial  1 week  2 weeks  1 month  2 months | Very Good | MDC = 0.84-6.76 | ? |
| *Battery Gait Assessment* | | | | | | |
| Howell et al., 2019 | n = 15 | ~16 years | ~7 days | Very Good | SEM = 0.05 | ? |
